# Co-existence of beta-lactamases in clinical isolates of *Escherichia coli* from Kathmandu, Nepal

**DOI:** 10.1186/1756-0500-7-694

**Published:** 2014-10-07

**Authors:** Ram Hari Pokhrel, Badri Thapa, Rajesh Kafle, Pradeep Kumar Shah, Chanwit Tribuddharat

**Affiliations:** Department of Microbiology, St. Xavier’s College, Kathmandu, Nepal; Department of Microbiology, Kathmandu Medical College and Teaching Hospital, Kathmandu, Nepal; Genesis Laboratory and Research, Kathmandu, Nepal; Department of Microbiology, Siriraj Hospital, Mahidol University, Bangkok, Thailand

**Keywords:** ESBL producing *Escherichia coli*, Carbapenemases, Clinical isolates, Integron element

## Abstract

**Background:**

The trend of extended-spectrum beta-lactamases producing *Escherichia coli* (ESBL-EC) is increasing in Nepal. Limited studies have been reported investigating ESBL types and carbapenemases in *E. coli*.

**Methods:**

A cross sectional study was conducted between June 2012 to January 2013 in Kathmandu Medical College and Teaching Hospital, Nepal. Non-repetitive clinical samples from out-patient department (OPD) and Intensive Care Units (ICU) were processed for bacteriological culture and identification of *E. coli.* Antibiotic susceptibility test, screening and phenotypic confirmation for ESBLs and carbapenemases and PCR (*bla*_CTX-M_, *bla*_SHV_ and *bla*_TEM_-type ESBLs, *bla*_VIM_, *bla*_IMP_ and *bla*_NDM-1_-type carbapenemases, and class 1 integron element integrase gene) were performed. Clones were resolved by PCR-Randomly Amplified Polymorphic DNA.

**Results:**

Out of 332 non-repetitive clinical specimens processed for culture and identification 160 (48.2%) were culture positive. Of which, 93 (58.1%) were *E. coli*. Of these, 24 (25.8%) were phenotypically confirmed as ESBL-EC and 3 (12.50%) of 24 ESBL-EC were carbapenemase producers. *bla*_CTX-M_-type ESBL was most common (23, 95.8%) followed by *bla*_TEM_ (7, 29.2%) and *bla*_SHV_ (3, 12.5%). *bla*_VIM_, *bla*_IMP_ and *bla*_NDM-1_ were present in 3, 2 and 2 ESBL-EC, respectively. Class 1 integron element was present in 18 (75.0%) ESBL-EC. Nine isolates possessed more than one type of beta-lactamases. Interestingly, all carbapenemase producers were isolated form ICU and co-existence of *bla*_CTX-M_, *bla*_SHV_, *bla*_TEM_, *bla*_IMP_, *bla*_VIM_ and *bla*_NDM-1_ beta-lactamases was documented in one ESBL-EC (EC104). All most all isolates had different RAPD patterns.

**Conclusions:**

For the first time in Nepal, high prevalence of *bla*_CTX-M_-type ESBL and co-existence of ESBLs and carbapenemases has been described. Continuous monitoring and surveillance and proper infection control and prevention practices will limit the further spread of these super-bugs within this hospital and beyond.

## Background

*Escherichia coli* is associated with numerous infections such as urinary tract infection, surgical site infection, skin and soft tissues infection, bacteraemia, pneumonia etc [1–3]. These infections are common among out-patient and Intensive Care Unit (ICU) admitted patients [4]. ICU patients are subjected to numerous invasive procedures and are susceptible to ICU acquired infections (IAI) and *Escherichia coli* is a common pathogen [5]. Extended-spectrum beta-lactam antibiotics-third generation cephalosporins- are commonly used for treating infections caused by *Escherichia coli* in Kathmandu Medical College and Teaching Hospital (KMCTH), Nepal. These antibiotics are less effective as Extended-spectrum beta-lactamase producing isolates of *E. coli* (ESBL-EC) is increasing in this institution [6]. Carbapenems are current choice for treating the infection caused by ESBL-EC however, emergence of carbapenem-resistant isolates has also been noticed [7]. Hence, the successful treatment outcome of *E. coli* infections is seriously tempered by these ESBL- and carbapenem-resistance.

Increase trends of ESBL and carbapenem-resistance over the past two decades has been noticed globally and also in Nepal [6, 8, 9]. Several variants of ESBLs; TEM, SHV and CTX-M have been described however; there is paucity in studies of ESBL and carbapenemases types from this institution and Nepal. This study aims to detect common ESBLs (*bla*_TEM_, *bla*_SHV_, and *bla*_CTX-M_) and carbapenemases (*bla*_IMP_, *bla*_VIM_*,* and *bla*_NDM_) in ESBL-EC isolated from Kathmandu Medical College Teaching Hospital, Nepal. Here, we describe high prevalence of *bla*_CTX-M_ type ESBL and carbapenemases producing *E. coli* and co-existence of ESBLs and carbapenemases in these isolates.

## Methods

### Specimens, inclusion criteria and identification of *E. coli*isolates

Non-repetitive clinical specimens (mid-stream urine, pus, discharge from surgical wound, endotracheal secretions, sputum, catheter tips etc.) received as part of standard patient care investigation from Intensive Care Unit (ICU) and out-patient department (OPD) in Kathmandu Medical College and Teaching Hospital between June 2012 to January 2013 were processed for culture and antibiotic susceptibility testing from patients attending OPD and admitted in ICU were included in the study. The patients already on antibiotics were excluded based on the history of antibiotics mentioned in the culture investigation form. *E. coli* isolates were isolated and identified using standard microbiological technique [5].

### Identification of *E. coli*isolates

All specimens were cultured on MacConkey and blood agar and incubated overnight at 37°C in the department of microbiology, KMCTH using standard microbiological technique [10]. On grown lactose fermenting colonies biochemical tests was performed to identify *E. coli.*

### Anti-microbial susceptibility technique

Kirby-Bauer disk diffusion test was performed on identified *E. coli* on Mueller-Hinton agar according to Clinical and Laboratory Standards Institute guidelines (CLSI) [11]. The following antibiotic disks procured from Hi Media Pvt. Ltd., India were used; ampicillin (20 μg) nalidixic acid (30 μg), amoxycillin (30 μg), amoxycillin-clavulinic acid (30 μg/10 μg), cefepime (30 μg), cefotaxime (30 μg), ceftriaxone (30 μg) and ceftazidime (30 μg), amikacin (30 μg), tobramycin (30 μg), gentamycin (10 μg) and imipenem (10 μg).

### Screening and phenotypic confirmation of ESBL and carbapenemase producers

The screening for ESBLs producers were performed using cefotaxime (30 μg), ceftazidime (30 μg) and ceftriaxone (30 μg) and interpreted based on CLSI guidelines [11]. Phenotypic confirmation of ESBL producers were confirmed using ceftazidime disc (30 μg) alone and in combination with clavulanic acid (10 μg). Similarly, imipenem resistant *E. coli* were confirmed for carbapenemase production by modified Hodge test [11].

### Genotype confirmation of ESBLs and carbapenemases

Crude DNA was extracted from pure culture of *E. coli*. Briefly, fresh culture of the test organism was suspended in 500 μl of saline, vortexed, boiled for 10 minutes, cellular debris was removed by centrifugation at 11,000 rpm for 5 min and supernatant was used as DNA template for PCR analysis. PCR amplification of the drug resistance genes like *bla*_TEM_, *bla*_SHV_, *bla*_CTX-M,_*bla*_IMP_, *bla*_VIM_ and *bla*_NDM-1_ was performed using gene specific primers (Table 1) and amplification profiles described earlier [12–14]. The PCR was performed in Genesis Laboratory and Research, Kathmandu, Nepal.

**Table 1:** **Primers used in this study**

Name of the primer	Target	Amplicon (bp)	Sequence 5′-3′
TEM1F	*bla*_TEM_	864	ATGAGTATTCAACATTTCCG
TEM1R			CTGACAGTTACCAATGCTTA
SHVF	*bla*_SHV_	865	GGTTATGCGTTATATTCGCC
SHVR			TTAGCGTTGCCAGTGCTC
CTX-MA1	*bla*_CTX-M_	544	*SCSATGTGCAG^≠^YACCAGTAA
CTX-MA2			CCGC^¥^RATATGRTTGGTGGTG
IMP F	*bla*_IMP_	569	TTGCCAGATTTAAAAAT
IMP 003			ACCAGTTTTGCCTTACCATA
VIM F	*bla*_VIM_	551	GTCTACCCGTCCAATGGTCTCA
VIM R			AGCAAGTCTAGACCGCCCG
NDM-1 F	*bla*_NDM-1_	593	GGTTTGGCGATCTGGTTTTC
NDM-1 R			CGGAATGGCTCATCACGATC
IntI1F	*intI1*	471	AAGGATCGGGCCTTGATGTT
IntI1R			CAGCGCATCAAGCGGTGAGC

Note: *S = G or C, ^≠^Y = C or T, ^¥^R = A or T.

### Controls

ESBL negative *E. coli* (ATCC 25922), ESBL positive *K. pneumoniae* (ATCC 700603) and Imipenemase producing *Pseudomonas aeruginosa* were used as controls in disk diffusion test, screening and confirmation tests. Multiple strains of *P. aeruginosa* genetically characterized to produce TEM, CTX-M, SHV, IMP, VIM and NDM-1 were used as positive controls for PCR.

### Clonal analysis

Polymerase Chain Reaction-Randomly Amplified Polymorphic DNA (PCR-RAPD) was to study the clonal nature of these isolates as described previously [13].

### Data analysis and ethical approval

The data is presented in frequency and percentages. The study was approved by the Institutional Ethical Review Board of Kathmandu Medical College and Teaching Hospital, Kathmandu, Nepal.

## Results

During the study period, 332 non-repetitive clinical samples [n = 292 (OPD) and n = 40 (ICU)] were processed and 160 samples (48.2%) [n = 130 (OPD) and n = 30 (ICU)] were culture positive. These specimens were received from urinary tract infections (110, 68.8%), respiratory tract infections (19, 11.9%), surgical site infection (16, 10.0%), skin and soft tissue infection (12, 7.5%) and blood stream infection (3, 1.9%) and *E. coli* was predominant pathogen (93, 58.1%). *E. coli* were derived mostly from urinary tract infection (80, 86.0%). Of which, 24 (25.8%) were phenotypically confirmed as ESBL-EC among which 10 ESBL producing isolates were from ICU. All ESBL-EC were found to be resistant to nalidixic acid, amoxycillin, cefepime, cefotaxime, ceftriaxone and ceftazidime. ESBL-EC were also resistant to amoxicillin-clavulinic acid (23, 95.8%), cotrimoxazole (21, 87.5%), imipenem (3, 12.5%), gentamicin (2, 8.3%), amikacin (1, 4.2%) and tobramycin (1, 4.2%).

*bla*_CTX-M_ was the most prevalent ESBLs (23, 95.8%) followed by *bla*_TEM_ (7, 29.2%) and *bla*_SHV_ (3, 12.5%) (Table 2). Among ESBL-EC, 3 (12.5%) (EC100, EC104 and EC107) isolates were screened and confirmed as cabapenemase producers. All these 3 isolates were from ICU. *bla*_VIM_ was present in all of these isolates , *bla*_NDM-1_ was present in 2 isolates (EC100 and EC104) and *bla*_IMP_ was present in 2 isolates (EC104 and EC 107). Strikingly, carbapenemase harboring isolates were found to contain more than one resistant gene under the study. Co-existence of ESBLs and carbapenemases among ESBL-EC was variable (Table 2). An EC104 harboured all resistant genes investigated. Class 1 integron element was prevalent among the ESBL producers (18, 75.0%).

**Table 2:** **Distribution of ESBL, carbapenemase and** ***IntI1 in*** **ESBL producing** ***E. coli***

ESBL producing *E. coli*	*bla*_CTX-M_	*bla*_SHV_	*bla*_TEM_	*bla*_NDM-1_	*bla*_IMP_	*bla*_VIM_	*IntI 1*
EC17, -23, -42, -103, -105, -106, -108, -205, -208,-209	+	-	-	-	-	-	+
EC206, -207, -210, -211	+	-	-	-	-	-	-
EC98, -102	+	+	-	-	-	-	+
EC202, -204	+	-	+	-	-	-	+
EC203, -212	+	-	+	-	-	-	-
EC100	+	-	+	+	-	+	+
EC104	+	+	+	+	+	+	+
EC107	+	-	+	-	+	+	+
EC201	-	-	-	-	-	-	+

**Figure 1:**
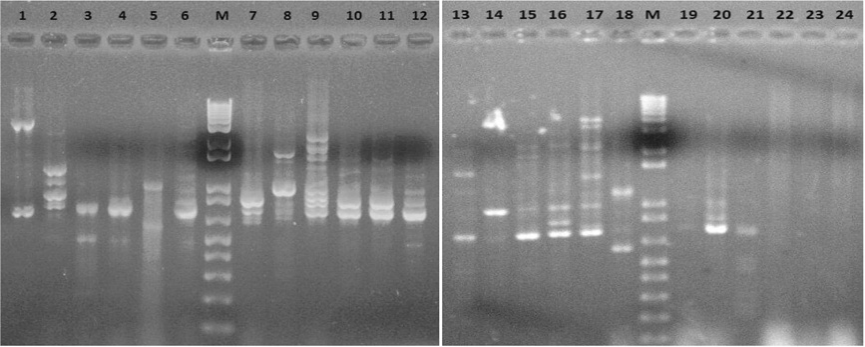
**Gel electrophoresis of PCR amplicons of RAPD patterns.** Lane M: Molecular weight marker (1 kb+ Invitrogen). Lane 1-24: E.coli isolates, EC17, EC23, EC42, EC98, EC100, EC102, EC103, EC104, EC105, EC106, EC107, EC108, EC201, EC202, EC203, EC204, EC205, EC206, EC207, EC208, EC209, EC210, EC211 and EC212.

All isolates of *E. coli* were subjected for RAPD. The isolates showed different RAPD patterns. Isolates EC106 & EC107 possessed similar RAPD patterns and rest of the isolates had individual RAPD patters (Figure 1).

## Discussion

*E. coli* is responsible for numerous infections and is frequently involved in sepsis and other infections in OPD and critically ill patients in Intensive Care Units (ICU) [5, 15]. The emergence of ESBL producing *E. coli* (ESBL-EC) is a real challenge for infectious disease medicine as these bugs are increasingly detected worldwide particularly in ICU [15, 16]. ESBL-EC infections ultimately results in unavoidable treatment outcomes and increases the cost in patients. In this study, 58.1% (93/160) of the infections were caused by *E. coli.* Of these infections, 25.8% (24/93) and 3.2% (3/93) were caused by ESBL-EC and carbapenemase producing *E. coli*, respectively. Frequency of isolation of *E. coli* is also common in ICUs elsewhere however infections due to ESBL-EC and carbapenemase producer vary among different geographical regions [5, 17]. Since 2000, the already ubiquitous *E. coli* has emerged as major ESBL producing organism. In 2007, already 79% of *E. coli* isolates collected in India were positive for ESBLs, with almost identical prevalence in hospital and community [18], 55% in China and 50.8% in Thailand [17]. ESBL-EC in ICU are increasing than general wards and out patients in this institution which is worrisome [6].

We have detected a variety of beta-lactamases among the isolates of *E. coli* namely *bla*_CTX-M_, *bla*_TEM_, *bla*_SHV_-type enzymes. The *bla*_CTX-M_ type was the most prevalent ESBL (n = 23). The incidence of this enzyme surpasses those of *bla*_TEM_ and *bla*_SHV_-type ESBLs in most locales worldwide [19] and also in our study. The wider spread of *bla*_CTX-M_ is also due to over use of third generation cephalosporins which has selected these strain. Some of the *bla*_CTX-M_ types are also associated with mobile genetic elements like class 1 integron element which contributes to its wider spread [20]. This was also evident in this study as 18 out of 23 *bla*_CTX-M_ positive isolates possessed class 1 integron element. However, the location of *bla*_CTX-M_ in class 1 element was not studied and needs further investigation. One of the isolate was ESBL-EC on screening and phenotypic test but didn’t possessed CTX-M, TEM and SHV enzymes, other ESBLs (AmpC) or other mechanisms could be possible [21].

The prevalence of *bla*_CTX-M_, *bla*_TEM_ and *bla*_SHV_- type ESBLs in *E. coli* is variable across different cities, countries and regions [22]. The prevalence of *bla*_CTX-M_ and *bla*_SHV_ genes was reported as 83% and 28%, respectively in ESBL-EC in New York [23] and 22.7% and 9.1%, respectively in ESBL-EC in Turkey [24]. Similarly, prevalence of *bla*_CTX-M_ and *bla*_TEM_ was 11% and 50%, respectively in Pakistan [25]. However, the *bla*_CTX-M_ has displaced other ESBLs in this geographical location as demonstrated in this study and also in Eastern Europe, South America, Japan and India [26].

The important finding in the study was the co-existence of different ESBLs and carbapenemases in the same isolate. Of 24 ESBL-EC, 9 (37.5%) possessed more than one ESBLs. Study in Taiwan reported co-existence of two or more kinds of ESBL in 40.6% of ESBL-EC [27]. Similarly, co-existence of *bla*_CTX-M_ and *bla*_TEM_ was found in 52.6% of French ESBL-EC [28]. Co-existence of NDM-1 and OXA-76 has been described in *Klebsiella pneumoniae* isolates from Nepal [29]. Carbapenemase producers were found to harbor carbapenemases co-existing with ESBLs. Each of EC104, EC100 and EC107 possessed *bla*_CTX-M_ + *bla*_TEM_ + *bla*SHV + *bla*NDM-1 + *bla*_IMP_ + *bla*_VIM_, *bla*_CTX-M_ + *bla*_SHV_ + *bla*_NDM-1_+ *bla*_VIM_ and *bla*_CTX-M_ + *bla*_TEM_ + *bla*_IMP_ + *bla*_VIM_, respectively. This co-existence of 6 beta-lactamases in EC104 was confirmed by multiple amplifications which is unique ESBL-EC in Nepal and elsewhere. The presence of carbapenemases like; *bla*_NDM-1_, *bla*_IMP_ and *bla*_VIM_ and its co-existence with ESBLs like, *bla*_CTX-M_, *bla*_TEM_ and *bla*_SHV_ in *E. coli* will seriously limit present and future therapeutic options.

The study of variants of ESBL-types, their location in mobile genetic elements (plasmids and integron elements), and clonal analysis of ESBL-EC is required. PCR-RAPD is simple, easy, cost-effective and has short turn-around time to answer the clonal nature of the bacterial isolates. PCR-RAPD was performed to study the clonal nature of these isolates but none of the isolates possessing similar resistance genes were grouped into similar RAPD types. More robust tools like pulse field gel electrophoresis and multi-locus sequence typing would help to know the exact clonal nature of these isolates.

## Conclusion

The high prevalence of *bla*_CTX-M_-type ESBL and co-existence of ESBLs and carbapenemases were noted in ESBL-EC isolated from Kathmandu Medical College and Teaching Hospital OPD and ICU patients for the first time. Continuous monitoring of this ESBL-EC with nationwide study will shed light in its dissemination and strategy to prevent and control the further spread of these super-bugs.
